# DeepCGAN: early Alzheimer's detection with deep convolutional generative adversarial networks

**DOI:** 10.3389/fmed.2024.1443151

**Published:** 2024-08-29

**Authors:** Imad Ali, Nasir Saleem, Musaed Alhussein, Benazeer Zohra, Khursheed Aurangzeb, Qazi Mazhar ul Haq

**Affiliations:** ^1^Department of Computer Science, University of Swat, Swat, KP, Pakistan; ^2^Department of Electrical Engineering, Faculty of Engineering & Technology (FET), Gomal University, Dera Ismail Khan, Pakistan; ^3^Department of Computer Engineering, College of Computer and Information Sciences, King Saud University, Riyadh, Saudi Arabia; ^4^Department of Anatomy, School of Medical Sciences and Research, Sharda University, Greater Noida, UP, India; ^5^Department of Anatomy, Noida International Institute of Medical Sciences, Noida International University, Greater Noida, UP, India; ^6^Department of International Bachelor Program in Informatics and Computer Science and Engineering, Yuan Ze University, Taoyuan City, Taiwan

**Keywords:** GAN, CNN, Alzheimer's disease, deep learning, cognitive features

## Abstract

**Introduction:**

Alzheimer's disease (AD) is a neurodegenerative disorder and the most prevailing cause of dementia. AD critically disturbs the daily routine, which usually needs to be detected at its early stage. Unfortunately, AD detection using magnetic resonance imaging is challenging because of the subtle physiological variations between normal and AD patients visible on magnetic resonance imaging.

**Methods:**

To cope with this challenge, we propose a deep convolutional generative adversarial network (DeepCGAN) for detecting early-stage AD in this article. The DeepCGAN is an unsupervised generative model that expands the dataset size in addition to its diversity by utilizing the generative adversarial network (GAN). The Generator of GAN follows the encoder-decoder framework and takes cognitive data as inputs, whereas the Discriminator follows a structure similar to the Generator's encoder. The last dense layer uses a softmax classifier to detect the labels indicating the AD.

**Results:**

The proposed model attains an accuracy rate of 97.32%, significantly surpassing recent state-of-the-art models' performance, including Adaptive Voting, ResNet, AlexNet, GoogleNet, Deep Neural Networks, and Support Vector Machines.

**Discussion:**

The DeepCGAN significantly improves early AD detection accuracy and robustness by enhancing the dataset diversity and leveraging advanced GAN techniques, leading to better generalization and higher performance in comparison to traditional and contemporary methods. These results demonstrate the ecacy of DeepCGAN in enhancing early AD detection, thereby potentially improving patient outcomes through timely intervention.

## 1 Introduction

Alzheimer's disease (AD) is a neurodegenerative condition primarily affecting the elderly, characterized by memory, behavioral, and cognitive impairments that disrupt daily life (1). This devastating disease is projected to have a staggering impact on global health in the coming decades. Epidemiological studies indicate a disturbing trend, with expectations of a fourfold surge in the worldwide prevalence of AD by 2050, potentially exceeding 100 million cases (2). The impending prevalence of AD raises critical concerns for individuals, families, and healthcare systems worldwide. The burden of AD extends beyond the individual, affecting the very fabric of society. Some studies employ mathematical modeling to forecast the trends and growth of AD, considering factors such as increasing life expectancy, shifting mortality patterns, and the prevalence of cardiovascular diseases. Unfortunately, these projections collectively suggest a growing proportion of the population will be impacted by AD in the future (3).

Detecting AD in its early stages is of paramount importance for effective intervention and treatment. AD diagnosis is a complex endeavor, demanding the accurate identification of different dementia subtypes (4). While the challenges are substantial, recent research highlights the central role of AD in dementia cases, constituting approximately two-thirds of all diagnoses (5). One of the pressing issues in AD management is the lack of effective pharmacological treatments in clinical practice. This shortfall has prompted a paradigm shift in therapeutic strategies, emphasizing the early-stage detection of AD as a promising avenue for intervention (6, 7). Identifying individuals in the early stages of cognitive decline or Mild Cognitive Impairment, whether stable or progressive, is pivotal for understanding high-risk populations and potentially delaying AD progression. The combination of the increasing prevalence of AD and the complexity of its diagnosis underscores the urgent need for advancements in early detection methods and comprehensive care strategies to address the growing global challenge of AD.

The AD research landscape has shifted significantly due to deep learning (DL) models, including stacked auto-encoders, recurrent neural networks, support vector machines, and convolutional neural networks (CNN). The bi-directional gated recurrent units (BiGRUs) layers consist of 2,048 units, with 1,024 units in each direction. The BiGRUs capture long-term temporal cues from the cognitive data, which is crucial for identifying patterns and changes indicative of early AD which have emerged as potent tools in this endeavor (8). However, limitations exist in feature quality, especially from image processing (9), requiring DL models adaptable to diverse data types. Simultaneously, generative adversarial networks (GANs), (which is a class of machine learning frameworks where two neural networks, a generator, and a discriminator, compete against each other to produce more accurate results) originally designed for images, have found their place in AD classification (9). DL models with GANs are proficient in classifying AD states and enhancing image-based AD tasks, like denoising images and precise brain segmentation (10, 11). These advances drive understanding, detection, and treatment of AD, a pressing neurodegenerative disease. Although, these architectures have made sufficient advancement in AD detection; however, these existing AD detection models have primarily focused on neuroimaging data, resulting in the underutilization of critical cognitive features. Moreover, temporal information, which is highly relevant for understanding AD progression, has been largely neglected in the literature. Additionally, the well-known challenge of training instability in these models remains a significant concern.

Existing models often struggle with limited dataset sizes and lack diversity, leading to overfitting and poor generalization. Traditional GAN-based methods, primarily designed for image data, fail to leverage cognitive data crucial for early AD detection. This article introduces a groundbreaking method for the early detection of AD—the deep convolutional generative adversarial network (DeepCGAN), which is an unsupervised generative model designed to leverage cognitive (clinical) data for AD detection. DeepCGAN addresses these issues by using a deep convolutional GAN framework to expand and diversify the dataset, generating high-quality synthetic data that improves detection accuracy and robustness. DeepCGANs generate high-quality synthetic medical images, crucial for augmenting limited datasets like MRI and PET scans and enhancing model generalization. They create diverse synthetic samples, augmenting training data in medical imaging where labeled data is scarce, improving model performance. DeepCGANs' convolutional layers learn complex features for accurate early Alzheimer's detection, and their flexibility across imaging modalities makes them versatile beyond disease detection, which makes DeepCGANs a powerful and effective choice for early AD detection.

To address the aforementioned gaps, the proposed model effectively incorporates and analyzes cognitive data, offering a more comprehensive understanding of AD. Also, the proposed model integrates temporal information using BiGRUs to capture long-term patterns and introduces mechanisms like gradient penalty and relativistic average loss to stabilize training, thereby enhancing the stability and reliability of AD detection with GANs. Operating through a dual structure, the Generator follows an encoder-decoder framework that takes cognitive data as input, while the Discriminator mirrors the architecture of the Generator's encoder. Moreover, the proposed model employs two distinct loss functions, Wasserstein and Relativistic loss, ensuring stable training and improved performance. The pivotal component of the model is the last dense layer, employing a softmax classifier to detect AD labels. The proposed DeepCGAN undergoes comprehensive training using cognitive data, demonstrating promising results in the early prediction of AD, achieving a remarkable 97.32% accuracy on cognitively labeled data from the ADNI dataset, surpassing recent state-of-the-art models. The contributions of this article include:

1. For detecting early-stage AD, this article proposes a DeepCGAN, an unsupervised generative model that extends the cognitive features of the data and its diversity by utilizing the GAN framework.
2. To optimize the detection performance of DeepCGAN, a novel convolutional encoder-decoder-based GAN is proposed and trained on the cognitive features.
3. Our comprehensive experiments on the ADNI dataset show that the proposed DeepCGAN performs better in detecting early-stage AD compared to start-of-the-art models.

The remainder of this article is structured as follows: Section 2 reviews related work. Section 3 presents the proposed DeepCGAN. Section 4 describes the experimental setup, and Section 5 discusses the evaluation of the proposed model. Finally, Section 6 concludes the article.

## 2 Related work

The current gold standard for detecting and prognosing neurodegenerative AD relies on clinical assessments of symptoms and their severity. However, early disease detection before clinical symptoms manifest is critical for effective disease management and timely therapeutic intervention. Research indicates that analyzing structural and functional changes in patients during the early stages of AD can provide valuable insights (12). Machine learning approaches offer a rapid and robust means to interpret medical examinations, aiding in the early detection of AD. Early detection is paramount, allowing for proactive intervention and potentially improving patient outcomes. Machine learning enhances the diagnostic process by uncovering subtle patterns and anomalies that may precede clinical symptoms. It transcends the limitations of conventional clinical assessments, which often rely on symptomatic markers that become evident at later disease stages. Integrating machine learning into AD detection represents a paradigm shift, emphasizing the significance of early and accurate diagnosis in transforming AD research and treatment strategies.

CNNs are deep learning models (13) known for their ability to extract complex patterns (14–16). They excel in body part segmentation, surpassing traditional methods like logistic regression and support vector machines (17). CNN-based computer-aided diagnosis (CAD) systems are effective in neurodegenerative disease detection (18). In AD detection, methods combining the dual-tree complex wavelet transform with neural networks show promise (19). Architectures like GoogleNet and ResNet deliver strong results in distinguishing healthy subjects from those with AD and mild cognitive impairment (20). LeNet-5 has been effectively employed for AD vs. normal control (NC) brain classification (21). Hosseini et al. extended previous work by proposing a Deeply Supervised Adaptive 3D-CNN (DSA-3D-CNN) for AD prediction (22). They trained this model on the CAD-Dementia dataset without skull stripping preprocessing and rigorously evaluated its performance through 10-fold cross-validation. In addition to CNNs, ensemble learning (EL) has proven invaluable in the detection and prognosis of neurodegenerative diseases. Given the often limited availability and the inherent 3D nature of medical imaging data, training classifiers can be a challenge (23). EL offers a promising solution by combining the strengths of multiple trained models, making it particularly useful for classification tasks involving heterogeneous datasets. To harness the power of ensemble learning, individual classifiers are trained on various subsets of the data and subsequently combined. EL with bootstrapping techniques becomes especially beneficial when relevant data is scarce, such as cognitive features. Additionally, when dealing with limited data, common practices include data augmentation to enhance the performance of ensemble models. This combined approach of CNNs and ensemble learning offers a robust and adaptable framework for tackling the complexities of neurodegenerative disease detection and prognosis.

GANs are a prominent method for enhancing imaging data by creating synthetic data that competes with a discriminator aiming to distinguish real from synthetic data (24). When generative networks excel, they can replicate data based on the inherent structure of real data. In the field of medical imaging, GANs have found success in tasks like MRI and CT reconstruction and unconditional image synthesis (25, 26). Furthermore, GANs exhibit a wide array of applications in AD-related image processing. They are proficient in denoising low-dose positron emission tomography (PET) scans to yield high-quality images (10, 11, 27). Accurate brain image segmentation, facilitated by GANs, aids in locating features critical for AD diagnosis and research across various image modalities (28–30). Despite the promise of GANs in AD image processing, the existing models for detecting AD have predominantly centered around neuroimaging data, leading to the insufficient utilization of vital cognitive features. Furthermore, the valuable temporal dimension, crucial for comprehending the progression of AD, has been notably overlooked in the existing literature. Additionally, the persisting issue of training instability in these models continues to pose a noteworthy challenge.

## 3 Materials and methods

The DeepCGAN model, proposed in this study, is designed for AD detection. It leverages a Generative Adversarial Network architecture, specifically tailored to the analysis of cognitive features and temporal information, which are often overlooked in existing AD detection models.

### 3.1 Generative adversarial networks

**Figure 1:**
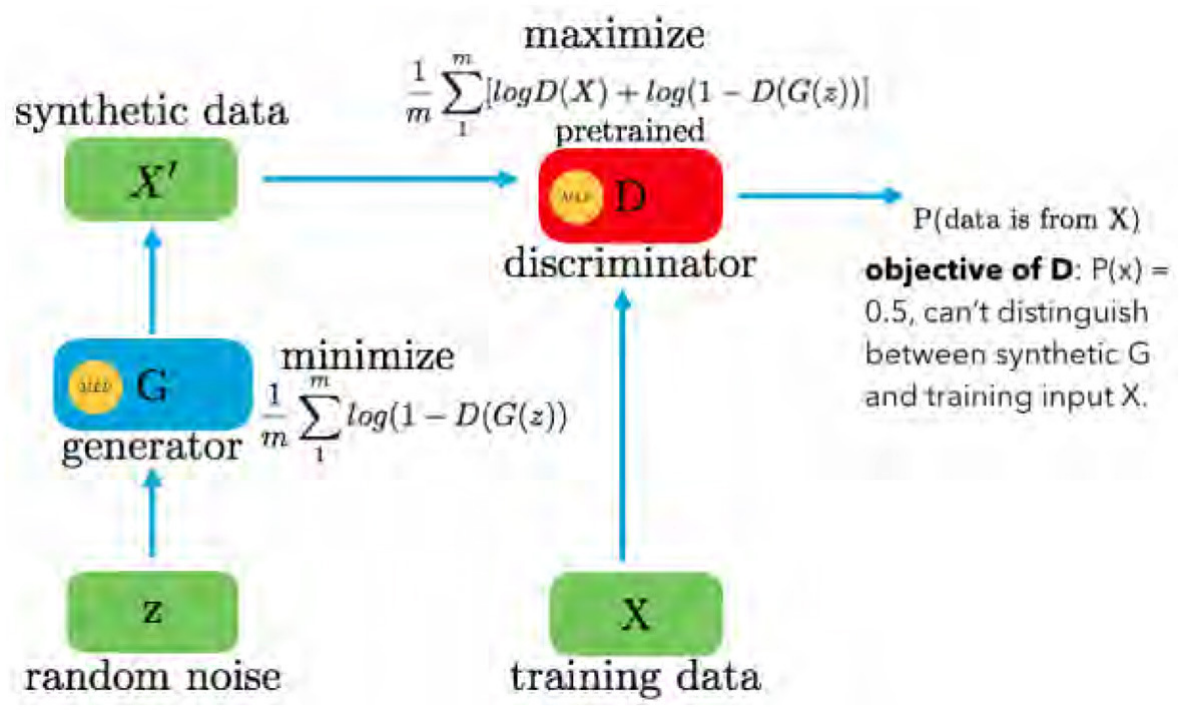
GAN framework with generator and discriminator.

GAN is a fundamental architecture in machine learning, composed of two primary components: the Generator *G*(*z*) and the Discriminator *D*(*x*), as shown in Figure 1. The GAN framework is designed for generative tasks, aiming to produce synthetic data that closely resembles real data distributions. The Generator *G*(*z*) is responsible for creating new data samples. It takes random noise *N*(*z*) as input, typically drawn from a uniform or normal distribution. Through a learned transformation process, the Generator generates data that mimics real training data. This process relies on adjusting internal parameters to produce data samples that are increasingly realistic.

The Discriminator *D*(*x*) acts as an adversary to the Generator. Its primary role is to differentiate between genuine data from the training set and data generated by the Generator. The Discriminator evaluates each input and assigns a probability score, indicating the likelihood of the input being real. If an input is genuine, *D*(*x*) approaches 1, whereas if it is generated, *D*(*x*) tends toward 0. The GAN operates as a two-player minimax game, optimizing the value function *V*(*G, D*). The objective function is given in Equation 1:


(1) $$\begin{array}{l} \underset{G}{\min}\underset{D}{\max}V\left(G,D\right)\;={\text{𝔼}}_{x\sim P_{\text{data}}\left(x\right)}\left[\log D\left(x\right)\right]\\ \;+{\text{𝔼}}_{z\sim P_{z}\left(z\right)}\left[\log\left(1-D\left(G\left(z\right)\right)\right)\right] \end{array}$$

Here, *D*(·) provides the probability that a given sample belongs to the training data *X*. The Generator aims to minimize log(1−*D*(*G*(*z*))), making *D*(*G*(*z*)) as high as possible, essentially fooling the Discriminator into considering *G*(*z*) as real data. Conversely, the Discriminator seeks to maximize *D*(*X*) and 1−*D*(*G*(*z*)), driving its optimal state toward *P*(*x*) = 0.5. In practice, GANs continually refine the Generator to produce data that is indistinguishable from real data, representing a powerful framework for generating synthetic data in various domains.

### 3.2 DeepCGAN for AD detection

**Figure 2:**
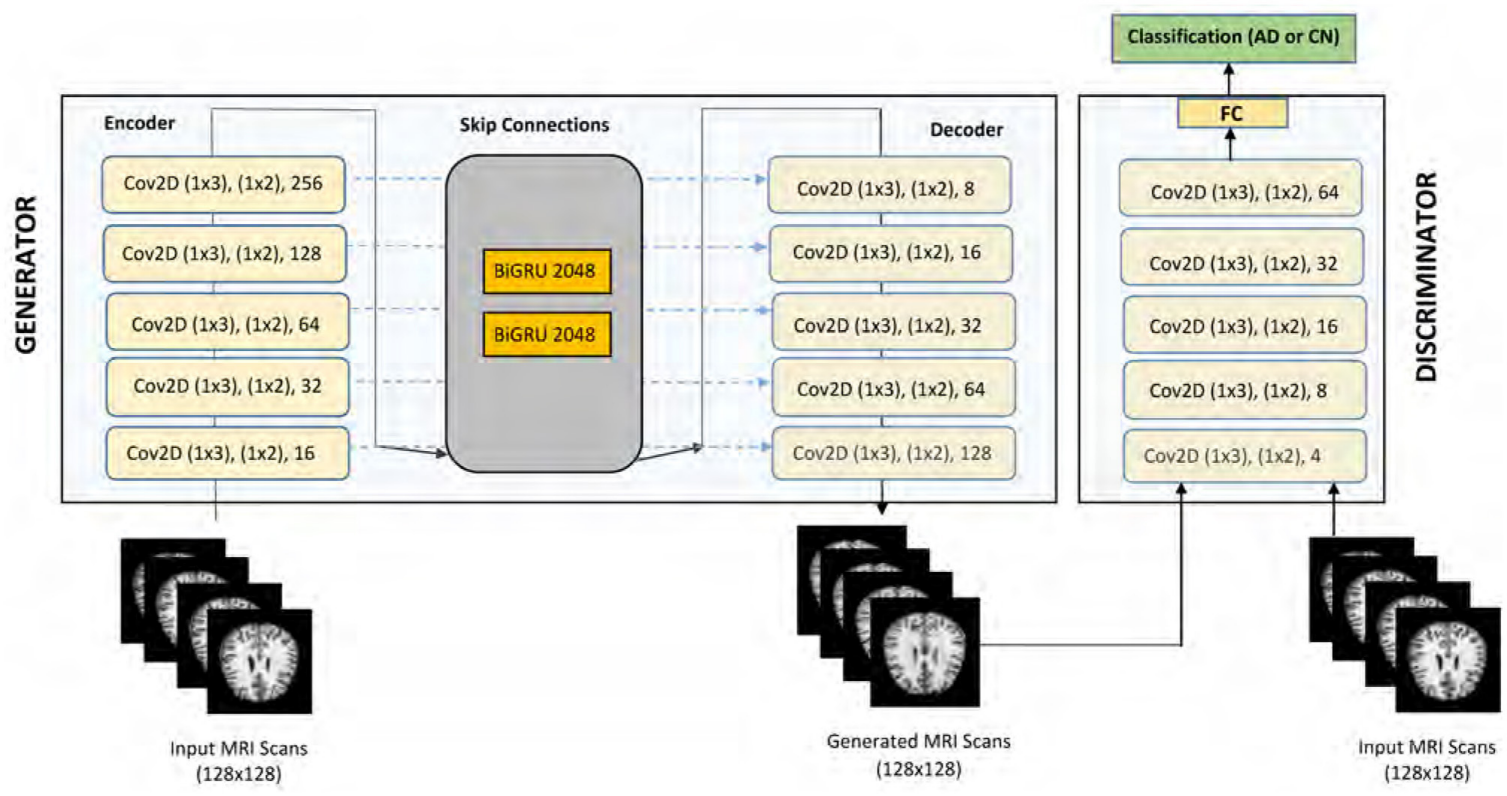
Proposed CNN-based GAN model (DeepCGAN).

The architecture of our proposed GAN model for AD detection is illustrated in Figure 2. This model is carefully designed to effectively utilize cognitive features in the detection process. The Generator component of our model is based on an encoder-decoder framework, optimized for processing cognitive features as inputs. The encoder in our model is designed to extract meaningful features from the input data through a series of convolutional layers. The encoder comprises five 2-D convolutional layers, strategically placed to extract local correlations within the input features. A reshape layer is employed to appropriately format the encoded features. These layers progressively downsample the input, capturing local correlations and essential patterns. Each convolutional layer is followed by batch normalization and Leaky Rectified Linear Unit (ReLU) activation functions to stabilize training and introduce non-linearity. Positioned in the middle of the Generator architecture, the BiGRU layers are crucial for capturing long-term dependencies and temporal dynamics in the cognitive features. Each BiGRU layer consists of 2,048 units (1,024 in each direction), enabling the model to learn bidirectional temporal patterns that are significant for early Alzheimer's detection.

The decoder mirrors the encoder's structure but performs the inverse operation. It utilizes deconvolutional (transposed convolution) layers to reconstruct the input data from the encoded features. The skip connections between corresponding layers of the encoder and decoder facilitate fine-grained feature integration, enhancing the model's ability to preserve important information during reconstruction. The Generator's primary function is to produce synthetic data that closely resembles the real cognitive feature data. By transforming random noise inputs through the encoder-BiGRU-decoder pipeline, the Generator learns to create realistic data samples that help augment the training set and improve the robustness of the Discriminator. The input to our DeepCGAN model consists of cognitive features derived from the ADNI dataset. The input to the model is a three-dimensional tensor with a batch size of 32, 50-time steps, and 128 features. Thus, the input shape is [32, 50, 128], specifically tailored to capture the temporal and cognitive aspects critical for Alzheimer's detection.

The data preprocessing steps include normalization and sequence padding to ensure uniform input dimensions. The preprocessing steps include: Normalization: The cognitive features are normalized to ensure consistent scales and improve model training stability. Padding: Sequences are padded to a fixed length (e.g., 50 time steps) to ensure uniform input dimensions across different samples. Handling Missing Values: Features with more than 40% missing values are removed. For the remaining features, missing values are imputed using appropriate statistical methods (e.g., mean imputation). In addition, two BiGRUs layers are thoughtfully inserted in the middle of the Generator architecture, which enhances the model's ability to capture long-term temporal cues from the cognitive data. This integration addresses a critical gap in existing models that primarily focus on neuroimaging data, thereby improving the detection of early AD. The decoder of our model mirrors the encoder's structure and consists of five 2-D deconvolutional layers, also known as transposed convolution layers. Batch normalization is consistently applied following each convolutional and deconvolutional operation. ReLU functions are used as activation functions within the hidden layers, while a sigmoid activation function is applied to the output layer. To facilitate fine-grained feature integration, we have incorporated skip connections within the Generator. These skip connections concatenate the outputs of each convolutional layer in the encoder with the corresponding inputs of the deconvolutional layers in the decoder. This design element enhances the model's ability to capture intricate feature cues effectively.

The Discriminator component, denoted as *D*, shares a similar structure with the encoder of the Generator. However, a flattened layer is introduced after the fifth convolutional layer to streamline feature processing. Finally, a fully connected dense layer with softmax activation is integrated into the Discriminator to enable classification tasks. Notably, the Discriminator provides two types of outputs, *D*(*y*) and *Dk*(*y*), with D(y) representing sigmoidal output and Dk(y) signifying linear output, linked by the sigmoid non-linearity function λ(*Dk*(*y*)) = *D*(*y*). The proposed GAN model for AD detection leverages cognitive features and exhibits a sophisticated architecture, comprising convolutional, deconvolutional, and recurrent layers, skip connections, and a dual-output Discriminator. These design innovations collectively contribute to the model's efficacy in AD detection. Most AD detection models predominantly focus on neuroimaging data, neglecting cognitive features. Our model efficiently incorporates and exploits these underutilized data sources. By including BiGRUs, our model accounts for long-term temporal cues, a crucial aspect often overlooked in AD progression analysis. The Discriminator's architecture, featuring dual output types and skip connections, introduces novel enhancements to improve the model's performance in distinguishing real and synthetic data.

The Discriminator is designed to differentiate between real and synthetic data. It shares a similar structure with the encoder and includes an additional fully connected layer with softmax activation for classification. The dual outputs of the Discriminator, D(y) and Dk(y), provide sigmoidal and linear outputs, respectively, enhancing the model's ability to distinguish between genuine and generated data. The DeeCGAN model is specifically tailored for Alzheimer's detection by focusing on cognitive features and temporal information, which are often underutilized in traditional models. By leveraging the strengths of DeeCGANs in generating realistic synthetic data and incorporating bidirectional GRUs for temporal analysis, our model is able to achieve high accuracy in early Alzheimer's detection.

### 3.3 Loss function

In the realm of GANs, choosing appropriate loss functions plays a pivotal role in achieving stable training and optimal performance. Our proposed GAN model incorporates and thoroughly investigates two distinct loss functions to determine the one that yields superior results.

#### 3.3.1 Wasserstein loss

The Wasserstein loss, denoted as *L*_*D*_ for the Discriminator and *L*_*G*_ for the Generator, offers significant advantages in stabilizing and enhancing the robustness of GAN models (18). The Wasserstein loss function is used to train the DeepCGAN model due to its ability to provide a smoother gradient, leading to more stable training. This stability is crucial for AD detection, as it ensures that the model effectively learns from the subtle and complex patterns in the cognitive data. These loss functions are defined in Equations 2–4:


(2) $$\begin{array}{l} L_{D}=-{\text{𝔼}}_{y\sim P\left(y\right)}\left[D_{k}\left(y\right)\right]+{\text{𝔼}}_{x\sim P\left(x\right)}\left[D_{k}\left(G\left(x\right)\right)\right], \end{array}$$

(3) $$\begin{array}{l} L_{G}=-{\text{𝔼}}_{x\sim P\left(x\right)}\left[D_{k}\left(G\left(x\right)\right)\right], \end{array}$$

(4) $$L_{GP}={\text{E}}_{\bar{y}\sim \bar{y},\tilde{y}}\left[{\left({\left\|\nabla_{\bar{y}}D_{k}(\tilde{y})\right\|}_{2}-1\right)}^{2}\right],$$

where $\nabla_{\bar{y}}D_{k}\left(ỹ\right)$ represents the gradient of the Discriminator output with respect to $\bar{y}$.

#### 3.3.2 Relativistic loss

The second loss function incorporated into our GAN model is the Relativistic loss. It computes the probability of real data features being classified as real and the probability of synthetic data features being classified as real. This is achieved by considering the difference between the Discriminator's outputs for real and synthetic input features. The loss functions for the Discriminator and Generator are given by Equations 5, and 6, respectively.


(5) $$\begin{array}{l} L_{D}=-{\text{𝔼}}_{\left(x,y\right)\sim P\left(x,y\right)}\left[\left(x,y\right)\left[\log\left(\upsilon D_{k}\left(y\right)-D_{k}\left(G\left(x\right)\right)\right)\right]\right], \end{array}$$

(6) $$\begin{array}{l} L_{G}=-{\text{𝔼}}_{\left(x,y\right)\sim p_{data}\left(x,y\right)}\left[\log\left(\nu_{D}^{k}\left(G\left(x\right)\right)-D_{k}\left(y\right)\right)\right] \end{array}$$

However, the the Relativistic loss in Equations 5 and 6 exhibits high variance, primarily when the Generator significantly influences the Discriminator. To address this, we consider the average loss functions for the Discriminator and Generator are given by Equations 7, and 8, respectively.


(7) $$\begin{array}{l} L_{D}=-{\text{𝔼}}_{y\sim P\left(y\right)}y\left[\log\left(D_{ȳ}\left(y\right)\right)\right]-{\text{𝔼}}_{x\sim P\left(x\right)}x\left[\log\left(1-D_{\bar{G\left(x\right)}}\left(x\right)\right)\right], \end{array}$$

(8) $$\begin{array}{l} L_{G}=-{\text{𝔼}}_{x\sim P\left(x\right)}x\left[\log\left(D_{\bar{G\left(x\right)}}\left(x\right)\right)\right]-{\text{𝔼}}_{x\sim P\left(x\right)}x\left[\log\left(1-D_{ȳ}\left(y\right)\right)\right], \end{array}$$

where *D*_ȳ_(*y*) and $D_{\bar{G\left(x\right)}}\left(x\right)$ represent the relativistic Discriminator outputs for real and synthetic data, respectively. Thus, our GAN model incorporates both Wasserstein and Relativistic loss functions, each with its distinct advantages. These loss functions are carefully chosen and utilized to optimize the model's training stability and performance in AD detection.

We selected the Wasserstein loss and Relativistic loss due to their efficacy in stabilizing GAN training and enhancing the quality of generated data. The Wasserstein loss addresses mode collapse and provides meaningful gradients for GAN convergence. The Relativistic loss improves the model's discriminative power by comparing real and generated data in a relativistic manner, aligning with the goal of distinguishing subtle differences in medical data. These loss functions balance sensitivity and specificity in Alzheimer's detection, with the Wasserstein loss ensuring high-quality synthetic data and the Relativistic loss enhancing the discriminator's accuracy and robustness.

## 4 Experiments

This section provides insights into the dataset, experimental settings, and an evaluation of the proposed model.

### 4.1 Dataset

We utilized the Alzheimer's Disease Neuroimaging Initiative (ADNI) dataset (20), consisting of three distinct stages. The ADNI dataset encompasses cognitive test scores and records of 5,013 instances, corresponding to 819 different AD patients. The cognitive features selected for this study include memory recall tests, attention assessments, and executive function evaluations, which are clinically relevant as they have been shown to be significant indicators of early cognitive decline associated with AD. Patients visited the clinic multiple times during clinical trials, resulting in new cognitive test scores generated and stored as additional records in the dataset for each visit. Among these records, there are 1,643 belonging to cognitively normal individuals and 3,370 related to AD patients. However, the dataset exhibited missing values and underwent initialization through an Iterative Imputer technique to impute the missing values using a round-robin method. This ensures that the most clinically significant features are retained and accurately represented in the dataset. The irrelevant features were removed during the data cleaning and preprocessing.

In the ADNI1 dataset, each record comprises 113 features. The data includes various cognitive assessments (e.g., MMSE scores, ADAS-Cog scores), biomarkers (e.g., cerebrospinal fluid biomarkers, amyloid-beta levels), and potentially neuroimaging features (e.g., MRI and PET scan data). These features are chosen for their relevance in assessing cognitive decline and AD progression. The input data is organized as a temporal sequence, capturing changes in cognitive features over time. This is crucial for modeling the progression of AD, which involves gradual cognitive decline. The dataset was divided into 80% for training and 20% for testing, resulting in 5,000 samples for training and 1,250 samples for testing. Some of these features had excessive missing values, prompting the removal of those with more than 40% missing values. The remaining features underwent initialization through an Iterative Imputer technique to impute the missing values using a round-robin method. After preprocessing, the final dataset comprised 4,500 samples. Additionally, the dataset contained features with varying value ranges, which were normalized to a range of 0–1 using the min-max scaling method. Primary filtering of cognitive features was performed using Pearson's correlation coefficient to identify those most correlated with AD diagnosis. Features with a correlation coefficient above a predefined threshold were selected for further analysis. Performance evaluation utilized metrics including Accuracy, Sensitivity, and F1-Score, which are computed by Equations 9, 10, and 11, respectively.


(9) $$\begin{array}{l} Accuracy=\frac{TP+TN}{TP+TN+FP+FN} \end{array}$$

(10) $$\begin{array}{l} Sensitivity=\frac{TP}{TP+FN} \end{array}$$

(11) $$\begin{array}{l} F1-Score=\frac{2TP}{2\left(TP+FP+FN\right)} \end{array}$$

Here, TP represents True Positives, TN stands for True Negatives, FP is False Positives, and FN represents False Negatives.

### 4.2 Network settings

The DeepCGAN model architecture for AD detection incorporates carefully chosen parameters to optimize performance across multiple metrics. The feature maps in the Generator's encoder are structured with fixed sizes of 16, 32, 64, 128, and 256 in successive convolutional layers, with specific kernel sizes and strides tailored to enhance feature extraction efficiency. The kernel size is set to (1,3) for the first 2D-Conv layer and (2,3) for subsequent layers, all with a stride of (1,2). Convolutional layers are utilized for their strength in extracting local patterns and hierarchical features from the cognitive data, which are essential for distinguishing subtle differences between normal and AD-affected individuals.Similarly, the BiGRU layers are configured with 2,048 units, with 1,024 units in each direction, split into forward and backward directions, operating over a fixed time step of 50. The BiGRUs were selected for their ability to capture long-term dependencies and temporal patterns in cognitive data, which are crucial for accurately modeling the progression of AD over time. For the Generator's decoder, these parameters are inversely set to reconstruct the input features faithfully. Moreover, the Discriminator (*D*) employs deconvolutional layers with gradually increasing feature maps from 4 to 64, designed to discriminate between real and generated samples effectively. The proposed AD detection models, incorporating these two distinct losses, undergo training and optimization using the Adam optimizer for 1,000 epochs, with a learning rate of 0.005 and a batch size of 32 samples. The combination of convolutional layers and BiGRUs in the DeepCGAN architecture leverages both spatial and temporal features, providing a robust framework for early AD detection by capturing complex patterns in cognitive data that simpler architectures might miss. This setup ensures robust optimization and convergence of the DeepCGAN model. To assess the performance of our proposed model, we conducted a comprehensive comparison with several other models, including DeciTree, RanForest, KNN, Linear Regression (LR), SVM, DNN, AdaBoost, and Adaptive Voting, utilizing various metrics such as Accuracy, Precision, Recall, and F1-Score.

## 5 Results and analysis

In this section, we present the results of our experiments and provide a comprehensive analysis of the findings.

### 5.1 Model performance comparison

Table 1 displays the results obtained from our proposed DeepCGAN model along with other DL models trained on similar cognitive features for detecting cognitive normal and AD. We measure the model's performance using Accuracy, Precision, Recall, and F1-Score as evaluation metrics. Notably, the results demonstrate that our proposed DeepCGAN outperforms all other competing models in terms of these metrics. The DeepCGAN achieved an Accuracy of 97.32%, Precision of 95.31%, Recall of 95.43%, and F1-Score of 95.61%, respectively. In contrast, the lowest-performing model, linear regression, achieved only 82.05% Accuracy, 81.83% Precision, and 81.45% F1-Score.

**Table 1:** Performance analysis using various measures for ADNI cognitive features dataset.

**DL model**	**Acc. (%)**	**Pre. (%)**	**Rec. (%)**	**F1-S. (%)**
DeciTree	88.93	88.93	88.93	88.93
RanForest	90.33	90.30	90.33	90.31
KNN	85.14	85.40	85.14	85.24
LR	82.05	81.83	82.05	81.45
SVM	85.84	85.68	85.84	85.71
DNN	90.53	90.67	90.53	90.59
AdaBoost	86.64	86.61	86.64	86.31
AdapVoting	93.92	93.89	93.92	93.89
DeepCGAN	97.32	95.31	95.43	95.61

**Figure 3:**
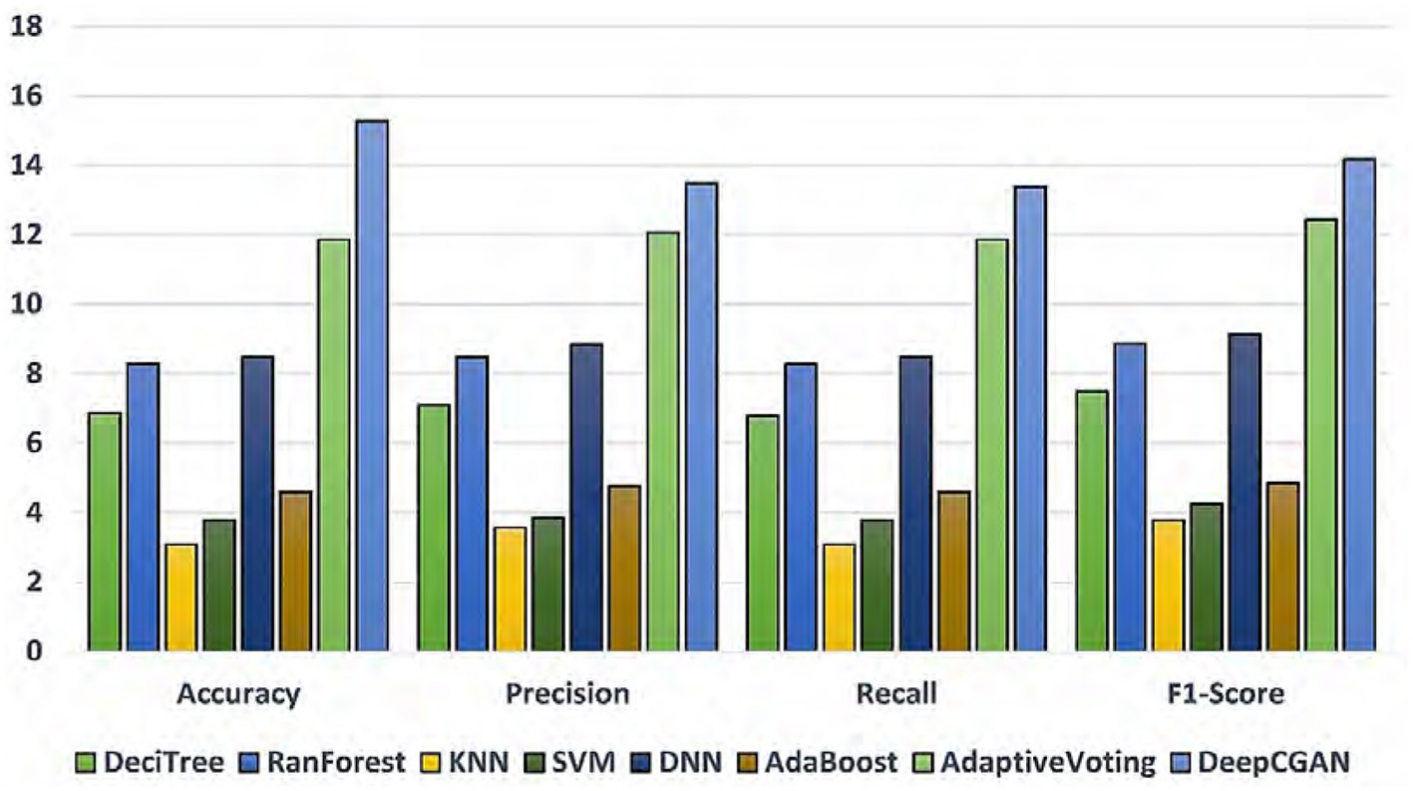
Percentage improvements in accuracy, precision, recall, and F1-score with linear regression as reference lowest model.

To highlight the improvements made by our proposed model, we chose linear regression as a reference model. DeepCGAN substantially improved Accuracy by 15.27%, Precision by 13.48%, and F1-Score by 14.16% compared to linear regression. Moreover, when compared to the second-best model, Adaptive Voting, DeepCGAN showed a 3.40% improvement in Accuracy. It also outperformed DNN and Random Forest by 6.79 and 6.99% in Accuracy, respectively, which is a significant performance gain. Furthermore, our DeepCGAN model demonstrated substantial improvements in Recall and F1-Score compared to competing models. The Recall increased from 88.93% (DeciTree) to 95.43% with DeepCGAN, and the F1-Score increased from 86.31% (AdaBoost) to 95.61%. These results signify the superior ability of DeepCGAN to correctly identify AD cases while minimizing false negatives. The errors are vastly improved over other models. To highlight the effectiveness of the proposed model, we present the overall improvements depicted in Figure 3. The linear regression is the reference model that has achieved the lowest performance among DL models. Figure 3 indicates the best performance of the proposed DeepCGAN.

### 5.2 Loss function analysis

**Figure 4:**
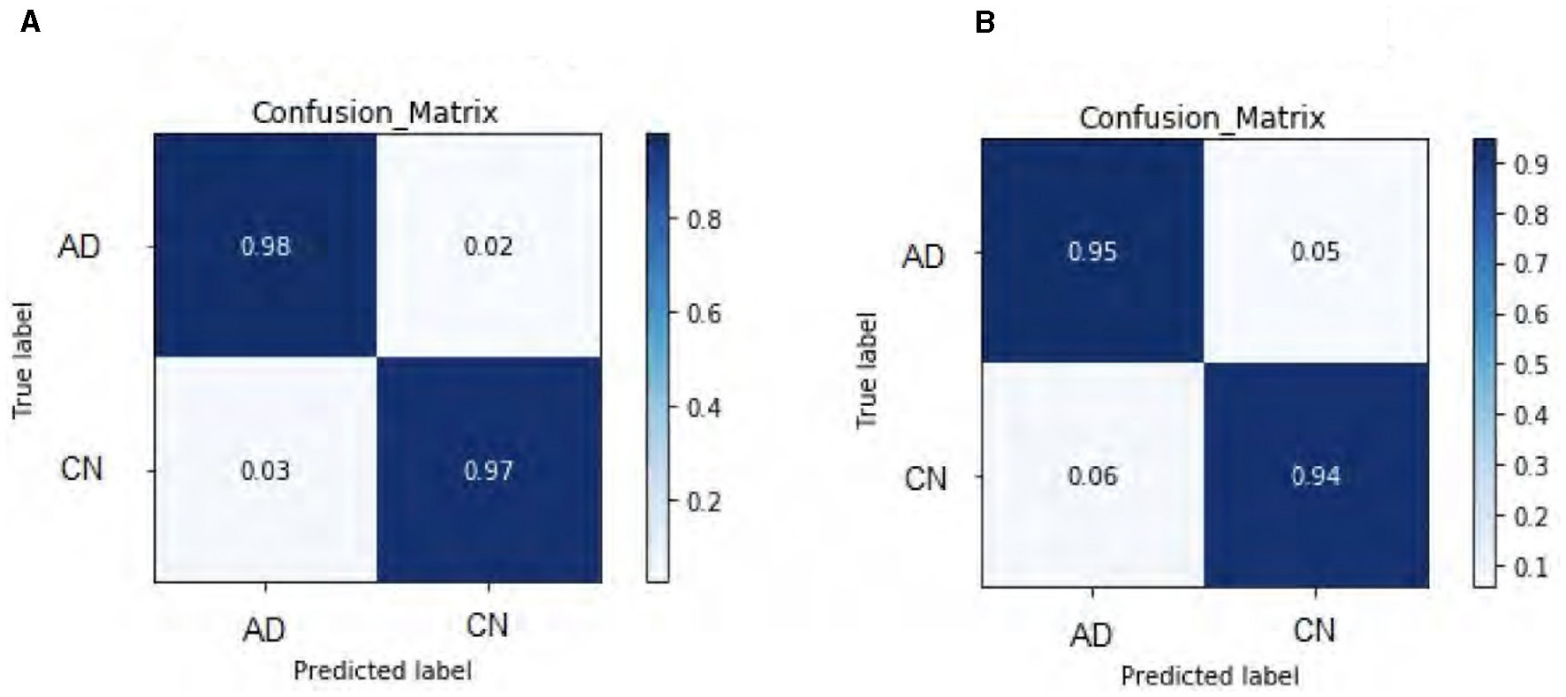
Confusion matrix heat-map for DeepCGAN. **(A)** Wasserstein loss. **(B)** Relativistic loss.

Our DeepCGAN model was trained and optimized using two different loss functions: Wasserstein and Relativistic loss. The Wasserstein loss function was chosen for its ability to provide a smoother gradient, thereby stabilizing the training process. This stability is particularly important for AD detection, where the model must accurately learn from subtle variations in cognitive data. Figure 4 presents the confusion matrices for both losses, revealing that the Wasserstein loss function results in better performance. Figure 4A illustrates the predicted labels when trained with Wasserstein loss, while Figure 4B shows the outcomes with Relativistic loss. It is evident that the model trained with Wasserstein loss provides more accurate predictions.

**Figure 5:**
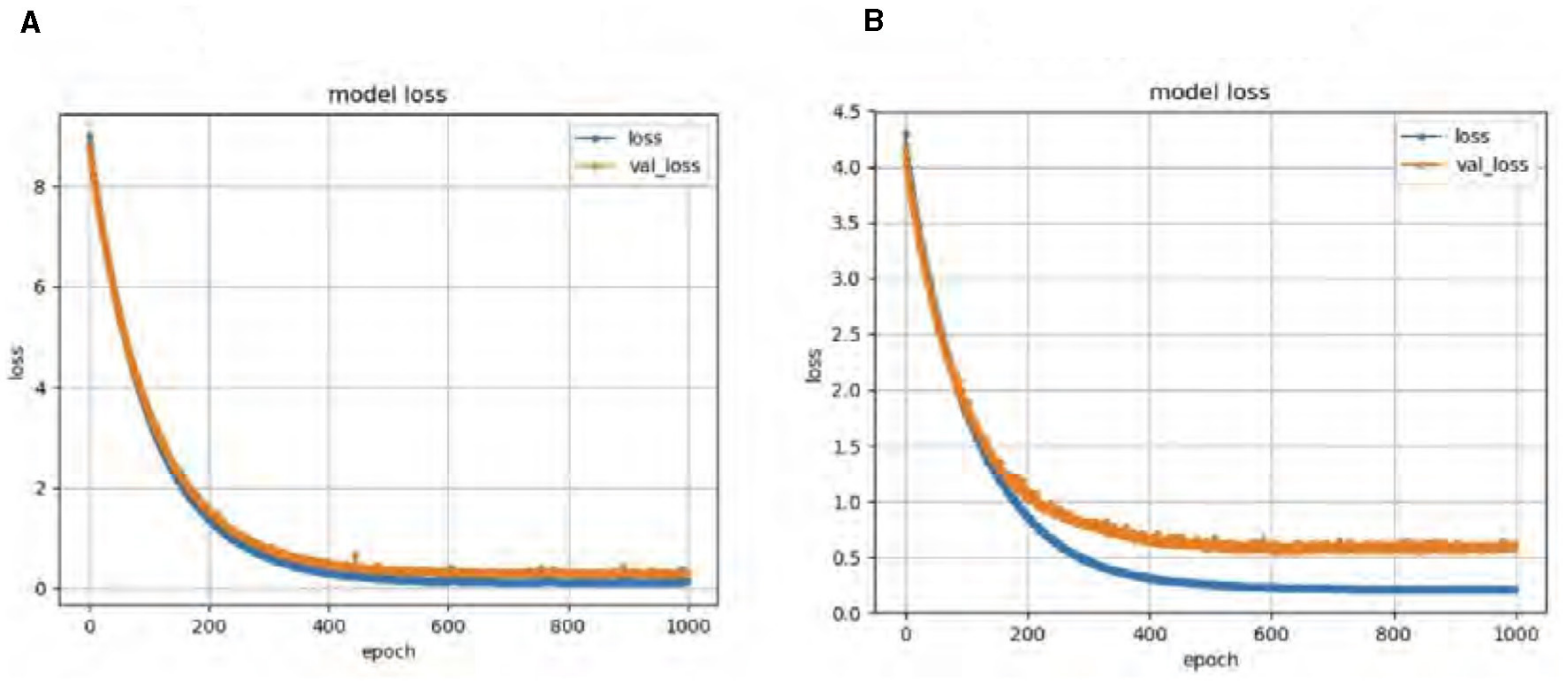
Loss curves for DeepCGAN. **(A)** Wasserstein loss. **(B)** Relativistic loss.

**Figure 6:**
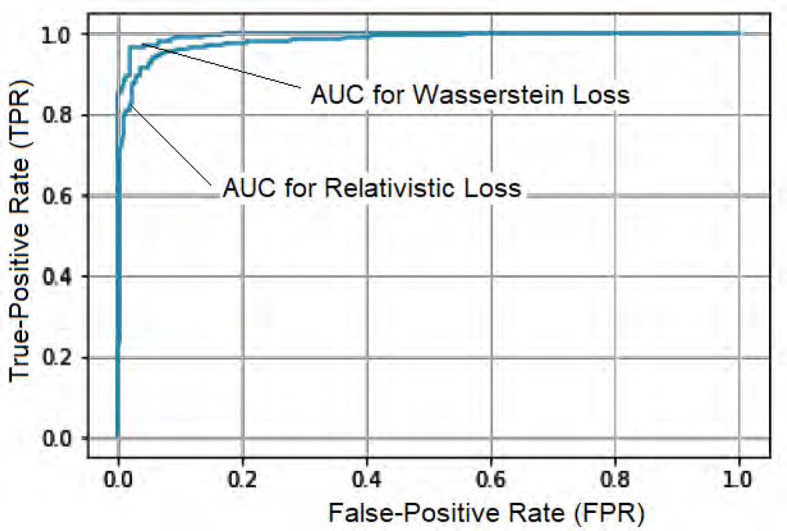
AUC curves for DeepCGAN.

To further assess the performance, we compared the model's Accuracy on training and validation data. The Wasserstein loss outperformed the relativistic loss by a significant margin, indicating faster convergence and better Accuracy. Figure 5 displays the loss curves over 1,000 epochs, illustrating the superior performance of the DeepCGAN model in achieving its detection task. We also evaluated the Area Under the Curve (AUC), which measures the model's ability to differentiate between labels. DeepCGAN exhibited higher AUC values compared to models trained with relativistic loss, further confirming its superior discriminatory capability. Figure 6 illustrates the AUC curves for both loss functions.

### 5.3 Comparison against state-of-the-art models

In this subsection, we compare our proposed DeepCGAN model with state-of-the-art (SOTA) models in the literature, including AlexNet (31), VGG-16 (32), GoogleNet (33), and ResNet (34), using cognitive features from the ADNI dataset. This comparison aims to benchmark the performance of DeepCGAN under similar experimental settings and datasets. Table 2 presents the results in terms of Accuracy, Precision, Recall, F1-scores, and AUC.

**Table 2:** Performance analysis (in %) for SOTA using ADNI Cognitive features dataset.

**Model**	**Accuracy**	**Precision**	**Recall**	**F1-score**	**AUC**
AlexNet	93.75	94.98	92.28	93.61	93.68
VGG-16	94.96	94.02	95.43	94.97	94.96
GoogleNet	91.73	90.20	93.50	91.82	91.79
ResNet	94.96	93.00	97.15	95.03	94.98
DeepCGAN	97.32	95.31	95.43	95.61	99.51

DeepCGAN surpassed all SOTA models in terms of Accuracy, achieving an Accuracy of 97.32%, which is a 5.59% improvement over GoogleNet. Similarly, Precision improved from 90.20% (GoogleNet) to 95.31%, reflecting a 5.11% boost in Precision. When compared to AlexNet, DeepCGAN demonstrated a 3.57% increase in Accuracy. Furthermore, DeepCGAN achieved the highest AUC among all models, with a 99.51% AUC, outperforming ResNet by 4.53% and VGG-16 by 4.55%, highlighting its superior discriminatory power. Regarding Recall, DeepCGAN exhibited substantial improvements over SOTA models except for ResNet, where the results were marginally lower. Specifically, the Recall increased from 92.28% (AlexNet) to 95.43% with DeepCGAN. The F1-Score achieved with GoogleNet was 91.82%. DeepCGAN's superior performance can be attributed to its novel architecture, which combines convolutional layers for effective feature extraction and BiGRUs for capturing temporal dependencies. This dual approach enables the model to detect subtle changes and patterns in cognitive data more accurately than models that rely solely on neuroimaging data or simpler architectures. Additionally, the use of GANs for data augmentation increases the dataset's size and diversity, enhancing the model's generalizability and robustness. The core innovation lies in expanding the cognitive features dataset and enhancing its diversity through GANs.

DeepCGANs significantly enhance Alzheimer's detection due to their ability to generate realistic synthetic images, crucial for augmenting limited MRI and PET scan datasets. Their deep convolutional layers extract complex features, improving diagnostic accuracy by capturing subtle disease indicators. Adversarial training refines synthetic images iteratively, ensuring they closely resemble real patient data. DCGANs' adaptability across imaging modalities and superior performance in comparative evaluations underline their transformative role in improving diagnostic accuracy and clinical outcomes for AD.

### 5.4 Comparison with existing techniques

In this section, we compare our proposed DeepCGAN model with a recently reported technique by Gill et al. (35) that used cognitive features for AD detection. Both studies utilized the same ADNI dataset, and the results are presented in Table 3. Our proposed DeepCGAN model outperformed the model proposed by Gill et al. (35) and Adaptive Voting using cognitive features from the ADNI dataset. DeepCGAN achieved the highest Accuracy of 97.32%, representing a 15.52% improvement over Adaptive Voting and a 3.4% improvement over Gill et al.'s technique for early AD detection. This improvement is due to its ability to generate synthetic data that closely resembles the actual cognitive features, thus reducing overfitting and improving the model's ability to generalize to new, unseen data.

**Table 3:** Performance comparison of the proposed model on ADNI dataset cognitive features.

**Model**	**Records**	**Accuracy**	**AUC**
Gill et al. (35)	600	81.80%	79.0%
AdaptiveVoting	5013	93.92%	99.3%
DeepCGAN	5013	97.32%	99.5%

## 6 Conclusion

In this study, we propose a novel convolutional encoder-decoder-based GAN for early AD detection using cognitive features. This model leverages a Generator module with Conv2D and Deconv2D layers in an encoder-decoder architecture to optimize Accuracy, Precision, Recall, F1-Score, and AUC metrics. Our experimental results demonstrate the superior performance of DeepCGAN, which significantly advances early AD detection, and outperforms several state-of-the-art models and benchmarks across various measures, achieving an outstanding 97.32% Accuracy compared to most other DL models in this study's SOTA comparison. Moreover, We find that using the Wasserstein loss is superior for training the proposed GAN. While our GAN excels, it is important to acknowledge the potential of SOTA DL models for early AD detection, which offer advantages over non-DL techniques like Gill's study. These DL models can expedite diagnosis, making them valuable tools in the detection of neurodegenerative diseases like Alzheimer's. The unique contribution of DeepCGAN lies in its novel use of GANs to enhance the dataset's size and diversity, coupled with a sophisticated architecture that integrates convolutional layers and BiGRUs. This approach significantly improves accuracy, precision, and overall performance metrics in detecting AD at early stages, demonstrating the model's superior capability in distinguishing subtle cognitive changes indicative of early AD. In the future, we aim to develop even more robust and streamlined DL models for detecting early and various stages of AD. Our proposed DeepCGAN model significantly advances early AD detection by leveraging a convolutional encoder-decoder-based GAN with Wasserstein loss, achieving superior performance metrics compared to SOTA models such as AlexNet, VGG-16, GoogleNet, and ResNet. This novel approach enhances the diversity and richness of cognitive features, resulting in a remarkable improvement in accuracy, precision, and discriminatory power, as demonstrated through comprehensive comparisons with existing techniques and models.

## Data Availability

Publicly available datasets were analyzed in this study. This data can be found here: https://adni.loni.usc.edu/data-samples/adni-data/.
